# The side effects of dopamine receptor agonist drugs in Chinese prolactinoma patients: a cross sectional study

**DOI:** 10.1186/s12902-022-01009-3

**Published:** 2022-04-11

**Authors:** Xiaoan Ke, Linjie Wang, Meiping Chen, Shanshan Liu, Na Yu, Lian Duan, Fengying Gong, Huijuan Zhu

**Affiliations:** Department of Endocrinology, State Key Laboratory of Complex Severe and Rare Diseases, Dongcheng District, Key Laboratory of Endocrinology of National Health Commission, Union Medical College Hospital, Chinese Academy of Medical Science and Peking Union Medical College, No. 1 Shuaifuyuan, PekingBeijing, 100730 China

**Keywords:** Prolactinoma, Dopamine receptor agonist drugs (DAs), Side effects, Impulse control disorders (ICDs)

## Abstract

**Background:**

Recently, side effects from Dopamine Receptor Agonist Drugs (DAs) in treating pituitary prolactinoma have raised widespread concern. This study explores the incidence and influencing factors of DAs-related side effects in Chinese prolactinoma patients.

**Methods:**

A cross-sectional study was conducted. 51 prolactinoma patients treated with DAs, 12 prolactinoma or pituitary microadenoma patients without DAs treatment, and 33 healthy controls were included. The Barratt impulsivity scale-11, Patient Health Questionnaire 9, and the ICD screening questionnaire were all used to evaluate the psychological and physical side effects of DAs. Clinical data of all subjects were collected from their electronic medical records.

**Results:**

The incidence of ICDs in the treated group, the untreated group, and control group was 9.8% (5/51), 16.7% (2/12), and 9.1% (3/33), respectively. In the treated group in particular, there were 1 patient (2%, 1/51), 2 patients (3.9%, 2/51), and 2 patients (3.9%, 2/51) with positive screening for punding, compulsive shopping, and hypersexuality, respectively. In terms of depression, the incidence of "minimal", "mild" and "moderate" depression in the treated group was 62.8% (32/51), 25.5% (13/51), and 5.9% (3/51), respectively. The incidence of physical symptoms was 51.0% (26/51) in the treated group and gastrointestinal symptoms were the most common symptoms (33.3%, 17/51). In addition, we found that the various parameters of DAs treatment had no association with the occurrence of physical symptoms or ICDs (all *P* > 0.05).

**Conclusions:**

Chinese prolactinoma patients treated with DAs had a lower incidence of ICDs (9.8%), while gastrointestinal symptoms were common. In this way, more attention should be paid to side effects, especially physical symptoms, in Chinese prolactinoma patients with DAs therapy during follow-up regardless of dose.

**Supplementary Information:**

The online version contains supplementary material available at 10.1186/s12902-022-01009-3.

## Introduction

Dopamine receptor agonist drugs (DAs) are the preferred treatment for pituitary prolactinoma, and they have been shown to reduce serum prolactin levels and shrink pituitary adenoma [1]. At present, the most commonly used drugs are bromocriptine and cabergoline [2]. These drugs bind to D2 dopamine receptors in the tuberoinfundibular area and play a role in inhibiting prolactin secretion and lactotroph cell proliferation [3]. As the binding of DAs to dopamine receptors is nonspecific [4], long-term use of DAs in patients with prolactinoma may result in side effects [5].

The most common side effects of DAs include nausea, orthostatic hypotension, and headache [6, 7], and rare side effects include valve reflux and fibrotic cardiac valvulopathy [8]. Psychosocial symptoms, such as mania, anxiety, and depression, have also been found to occur after DAs therapy [7]. In addition, impulse control disorders (ICDs) are another common side effects of DAs that are manifested in difficulty controlling impulsive behaviors, such as shopping, gambling, and sexual desire [9]. Recently, researchers have begun to focus more and more attention on the psychosocial side effects of DAs in patients with prolactinoma [7, 10, 11]. Besides, pituitary prolactinoma is the most common functional pituitary adenoma with a great number of patients treated with DAs [12]. However, there have been no reports on these side effects in Chinese prolactinoma patients with DAs treatment. Therefore, we conducted this study in order to investigate specifically the incidence and related factors of side effects associated with psychosocial and physical symptoms in Chinese prolactinoma patients receiving DAs therapy and potentially to provide a basis for better clinical practice.

## Subjects and Methods

### Subjects

A cross-sectional study was conducted. 51 patients with pituitary prolactinoma treated with DAs (≥ 3 months) from the pituitary clinic of Peking Union Medical College Hospital from December, 2020 to April, 2021 were included as our treated group. Patients with mental illness, a history of intracranial surgery, major disease, or failure to complete the questionnaire were excluded. We included 2 untreated hyperprolactinemia patients with pituitary microadenoma for follow-up observation, 2 untreated prolactinoma patients and 8 prolactinoma patients with DAs withdrawal greater than 1 year (5 patients for remission and 3 patients for pregnancy) as the untreated group. 33 healthy people were included as the control group. Additionally, we collected medical records and examination data for all subjects.

All subjects received a questionnaire after informed consent and this study was approved by the Ethics Committee of Peking Union Medical College Hospital. Our diagnostic criteria for prolactinoma were as follows: typical clinical presentation, such as amenorrhoea, lactation, and hyposexuality, combined with high serum prolactin levels and a positive MRI examination of a pituitary adenoma, or a previous diagnosis of prolactinoma [13].

### Questionnaires and Screening Criteria

We administered an ICDs screening questionnaire that has been used previously in existing research [9]. The questionnaire included four aspects: hypersexuality, compulsive shopping, punding, and pathological gambling. Hypersexuality was defined as a “yes” answer to item 9 in the questionnaire and a total score of >  = 2; compulsive shopping was defined as a total score of >  = 9; punding was defined as a total score of >  = 4; pathological gambling was defined as a “yes” to item 29 in the questionnaire and a total score of >  = 5.

In addition, we also administered Patient Health Questionnaire 9, which is a depression scale with 9 items. Patients were asked to report based on their experiences in the past 2 weeks, and the scale was divided into four subscales (not at all = 0, a few days = 1, more than half of the time = 3, and nearly every day = 4). The depression grades included minimal (< 5 points), mild (5 to 9 points), moderate (10 to 14 points), moderately severe (15 to 19 points), and severe (> = 20 points) [10].

The Barratt impulsivity scale-11 (BIS-11) was also used as an ICD screening tool and included 30 questions covering attentional impulsiveness, motor impulsiveness, and non-planning impulsiveness. Patients were asked to record the frequency of each item on a 4-point scale (rarely/never = 1, occasionally = 2, often = 3, and almost always/always = 4). A total score of >  = 60 was considered to be a positive screen for ICD in patients with prolactinoma [10].

According to the instruction, guidelines, consensus, and previous literature [6, 7, 13, 14], we classified side effects of DAs related to physical symptoms into diverse aspects that consisted of systemic and cutaneous symptoms, respiratory symptoms, gastrointestinal symptoms, cardiovascular symptoms, urinary symptoms, and psychological and neurological symptoms. All subjects received the questionnaire on side effects related to physical symptoms (see supplemental materials. If have, tick items of symptoms; if unlisted, tick item Others and write the symptoms in the blank).

### Data Collection

We collected the medical history of all patients, including the history of their present illness, DAs treatment history, duration of DAs therapy, and the maximum daily/weekly dose of DAs. We also collected laboratory results, including serum levels of prolactin, follicle-stimulating hormone, luteinizing hormone, progesterone, testosterone, and data from the patients’ pituitary MRIs (first visit and last follow-up).

### Statistical Analysis

All statistical analysis was performed using SPSS (version 23.0) and Prism software. We used a Kolmogorov–Smirnov test to identify the normality of variables, and non-parametric tests were used to compare the differences in continuous variables between groups. Fisher's exact test was used to compare the differences in sex ratio and the incidence of physical and mental symptoms between groups. Additionally, we used Spearman correlation analysis was used to explore the related factors of physical symptoms and psychological side effects of DAs in the treated group. We used a threshold of *P* < 0.05 to indicate a statistically significant test result.

## Results

### Basic Information for All Subjects

As shown in Table 1, we found no significant differences in sex ratio, age, or BMI between the treated and untreated groups. Compared with the control group, age (36.8 ± 11.7 vs. 30.2 ± 5.6 years, *P *< 0.05) and BMI (24.26 ± 4.36 vs. 21.12 ± 1.77 kg/m^2^, *P *< 0.05) were significantly increased in the treated group. Serum prolactin (PRL) levels were significantly lower in the treated group than in the untreated group (median PRL: 17.6 vs. 43.1 ng/ml, *P *= 0.003), and we found no significant differences in the ratio of microadenomas to macroadenomas or the maximum diameter of adenoma between the treated and untreated groups (*P *> 0.05). Of the 45 and 6 patients treated with bromocriptine and cabergoline, respectively, we found that two patients treated with cabergoline had switched medications due to bromocriptine-related side effects.

**Table 1:** The basic information for all subjects

Variable	Treated (*N* = 51)	Untreated (*N* = 12)	Controls (*N* = 33)
Gender(F/M)	39/12	10/2	27/6
Age(y)	36.8 ± 11.7	42.7 ± 8.9	30.2 ± 5.6*
BMI (kg/m^2^)	24.26 ± 4.36	23.53 ± 3.27	21.12 ± 1.77*
PRL (ng/ml)	17.6 (1.6, 417.0)	43.1 (14.4, 151.1) *	—
Micro/Macro	39/12	11/1	—
D-max(mm)	6.5 ± 3.6	6.1 ± 2.5	—
Treatment			
Bromocriptine (N)	45	—	—
Duration (m)	26 (3, 98)	—	—
Total dose(mg)	3412.5 (112.5, 39,067.5)	—	—
Max-dose(mg/d)	5.0 (1.3, 22.0)	—	—
Cabergoline (N)	6	—	—
Duration (m)	13 (3,59)	—	—
Total dose(mg)	179.7 (20.0, 560.0)	—	—
Max-dose(mg/w)	2.5 (1.3,4.0)	—	—

Micro/Macro is the ratio of microadenomas to macroadenomas. D-max is the maximum diameter of prolactinoma at the last follow-up

^*^compared with the treated group and having a *P* value < 0.05

### The side effects of dopamine receptor agonist drugs (DAs)

The screening results of psychological side effects are shown in Table 2. The incidence of ICDs (screened by a questionnaire from the Mayo Clinic [10]) was 9.8% (5/51) in the treated group, and there were 1 (2.0%), 2 (3.9%), and 2 (3.9%) patients with positive screening for punding, compulsive shopping, and hypersexuality, respectively in the treated group as well (Table 3). However, we found no significant difference in the incidence of ICDs among the three groups (all *P* > 0.05). In addition, the positive rates of BIS-11 screening in both the untreated and control groups were significantly higher than that in the treated group (49.0 vs. 83.3% and 49.0 vs. 78.8%, respectively, both *P* > 0.05). As for depression, the incidence of "minimal", "mild", and "moderate" depression in the treated group was 62.8% (32/51), 25.5% (13/51), and 5.9% (3/51), respectively, and there was no significant difference in the incidence of different levels of depression among the three groups (all *P* > 0.05).

**Table 2:** The psychological side effects of DAs

Variables	Treated N/Total (%)	Untreated N/Total (%)	Controls N/Total (%)
ICDs $	5/51 (9.8)	2/12 (16.7)	3/33 (9.1)
Punding	1/51 (2.0)	0/12 (0)	0/33 (0)
Pathological gambling	0/51	0/12	0/33
Compulsive shopping	2/51 (3.9)	2/12 (16.7)	3/33 (9.1)
Hypersexuality	2/51 (3.9)	0/12 (0)	0/33 (0)
BIS-11(> = 60 points)	25/51 (49.0)	10/12 (83.3) *	26/33 (78.8) *
Depression			
Minimal (< 5 points)	32/51 (62.8)	6/12 (50.0)	23/33 (69.7)
Mild (5–9 points)	13/51 (25.5)	6/12 (50.0)	9/33 (27.3)
Moderate (10–14 points)	3/51 (5.9)	0/12 (0)	1/33 (3.0)
Moderately severe (15–19 points)	0/51	0/12	0/33
Severe (> = 20 points)	0/51	0/12	0/33

^$^ means the results of a questionnaire from the Mayo Clinic

^*^compared with the treated group and having a *P* value < 0.05

**Table 3:** Basic information for patients with ICDs by the questionnaire from the Mayo Clinic

Variables	Patient 1	Patient 2	Patient 3	Patient 4
Gender	Female	Mele	Male	Female
Age (y)	22	30	37	35
BMI (kg/m^2)^	18.59	29.41	20.90	25.65
Basic PRL (ng/ml)	94.7	197.0	200.0	109.0
PRL (ng/ml)	1.6	26.3	35.6	15.5
LH (IU/L)	1.60	1.22	1.50	3.43
FSH(IU/L)	7.65	4.28	3.63	2.79
E2 (pg/ml)	40	17	16	147
P (ng/ml)	0.53	0.28	0.72	17.81
T (ng/ml)	0.52	2.03	2.20	0.66
Adenoma	Micro	Macro	Micro	Micro
D-max (mm)	—	10.6	9	—
Medication	Bromoc	Bromoc	Bromoc	Bromoc
Duration (m)	12	42	6	36
Total dose(mg)	1368.75	2934.38	450	2475
Max-dose (mg/d)	6.25	3.75	2.5	5
Side effects				
Physical symptoms	Non	Vomiting, Dry mouth, Constipation, Memory loss	Non	Fatigue, Asthenia, Nausea, Dizzy, Somnolence
Punding	No	No	No	Yes
Pathological gambling	No	No	No	No
Compulsive shopping	Yes	No	No	Yes
Hypersexuality	No	Yes	Yes	No
Depression	Mild	Mild	Minimal	Mild
BIS-11	72 points positive	67 points positive	52 points negative	76 points positive

D-max is the maximum diameter of prolactinoma at the last follow-up. Bromoc is short for bromocriptine

**Fig. 1:**
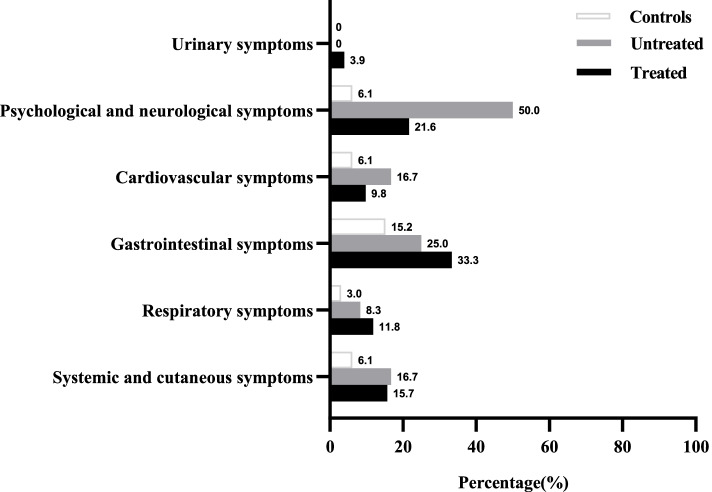
The side effects of DAs related to physical symptoms in patients with prolactinoma

The side effects related to physical symptoms in all three groups are shown in Fig. 1. The incidence of physical symptoms was 51.0% (26/51) in the treated group, and gastrointestinal symptoms were the most common type (33.3%, 17/51), mainly including loss of appetite (41.2%, 7/17) and nausea (35.3%, 6/17). In addition, one patient developed mild aortic insufficiency after treatment with cabergoline. However, we found no significant differences in the occurrence of symptoms in the treated and untreated groups (all *P* > 0.05).

Thus, the incidence of ICDs in our sample of Chinese prolactinoma patients treated with DAs was 9.8% and was not statistically different from the untreated or control group. Physical symptoms were common, with an incidence of 51.0%, and gastrointestinal symptoms were the most common type of physical symptoms.

### Factors Associated with the Incidence of DA-Related Side Effects

We further analyzed 45 patients treated with bromocriptine to explore factors associated with DA-related side effects, and our results showed that the occurrence of physical symptoms was related to the type of adenoma (*r* = 0.400, *P* = 0.008). Compared with patients with pituitary microadenoma, the incidence of physical symptoms in patients with pituitary macroadenoma was significantly higher [8/9 (88.9%) vs. 14/36 (38.9%), *P* = 0.010]. In addition, positive screening for hypersexuality was associated with gender (*r* = 0.379, *P* = 0.012). However, we found no significant difference in the incidence of hypersexuality between males and females (0/34 (0%) vs. 2/11 (18.2%), *P* = 0.056). Furthermore, the scores of BIS-11 scales were related to gender with higher scores in women (*r* = -0.325, *P* = 0.029). However, we found no significant difference in the positive rate of BIS-11 questionnaire screening for ICDs between males and females (*P* = 0.138). Interestingly, we did not find that parameters related to bromocriptine treatment, including duration of treatment, cumulative dose, and maximum daily dose, were significantly associated with physical symptoms, ICDs, or depression (all *P* > 0.05).

## Discussion

This study found that the incidence of ICDs in Chinese prolactinoma with DAs was low (9.8%, screened by a questionnaire from the Mayo Clinic). Specifically, 1 (2%), 2 (3.9%), and 2 (3.9%) patients had a positive screening for punding, compulsive shopping, and hypersexuality, respectively. Physical symptoms were common with an incidence of 51.0%, and gastrointestinal symptoms, which mainly included loss of appetite (41.2%) and nausea (35.3%), were the most common type of these. In addition, therapy-related parameters were not significantly associated with the occurrence of physical symptoms, ICDs, or depression.

Treatment with DAs can cause many different side effects, such as nausea, vomiting, orthostatic hypotension, and headache [6, 12, 15], that may be related to 5-HT1 receptors or D1 receptors. In particular, bromocriptine may have more side effects compared with cabergoline or quinoline, due to its shorter half-life and lower activity in D2 receptor activation [16, 17]. Consistent with previous studies [16, 17], we found that gastrointestinal symptoms were the most common physical side effects of DAs, accounting for 33.3% of them. These symptoms included loss of appetite (7/17, 41.2%), nausea (6/17, 35.3%), dry mouth (5/17, 29.4%), and constipation (5/17, 29.4%). Therefore, we recommend that DAs be started with a small dose that should be gradually increased and always taken with meals in order to reduce digestive symptoms [12].

Another common side effect of DA therapy is the onset of various ICDs, and these may be related to binding with the D3 dopamine receptor in the mesocorticolimbic area [18]. A series of studies have shown that the incidence of ICDs in prolactinoma patients treated with DAs is anywhere from 8% to 61.1% [9, 11, 19–21], while the incidence in our research was 9.8% (5/51), suggesting a wide range indeed. There may be two reasons for this.

First, the assessment tools used for ICDs in previous studies included the Minnesota Interview Questionnaire (MIDI) [19], the Modified ICDs Screening Questionnaire from Parkinson disease and restless leg syndrome patients (a questionnaire from the Mayo clinic) [9], the revised version of the Minnesota Impulsive Disorders Interview Questionnaire (MIDI-R) [20], the Barratt Impulsiveness Scale-11 (BIS-11) [11, 20], and the Questionnaire for Impulsive-Compulsive Disorders in Parkinson’s Disease (QUIP) [11]. Therefore, different assessment tools and screening criteria in various studies can lead to inconsistent ICDs screening results.

Second, studies across countries have shown that the incidence of ICDs in pituitary prolactinoma patients treated with DAs does vary: 10% (2/20) in Slovakia [19], 24.68% in the United States [9], 17% in Turkey [11], and 61.1% in Australia [21]. Our study firstly found that the incidence of ICDs was 9.8% in Chinese patients with prolactinoma. Therefore, the use of various assessment tools and the inherent diversity of differential populations may both lead to a difference in the observed incidence of ICDs. Hence current results need to be verified by further expanding the sample size and taking social, cultural, and other influencing factors into account.

We also found that gender plays a role in certain ICDs with males being the most vulnerable to hypersexuality, while females mostly develop compulsive shopping [7, 22]. Dogansen [11] et al. showed that the incidence of hypersexuality was the highest in males and significantly higher than that in females (14% vs. 3.2%, *P* < 0.001). Another study also showed that male pituitary prolactinoma patients who received DAs had a significantly higher incidence of hypersexuality (80.0% vs. 40.9%, *P* = 0.001) than a control group [21]. We observed that 2 patients with hypersexuality were both male and 2 patients with compulsive shopping were both females in this study, suggesting that gender differences do exist for certain ICDs. However, we found no significant difference in the incidence of hypersexuality and compulsive shopping between males and females, which may be related to our small sample size and the small number of ICD cases in this study. This too is an argument in favor of a larger future study.

It was worth mentioning that the treatment dose of DAs in Parkinson's disease patients was higher, and the occurrence of ICDs was dose-dependent [22–25]. However, the dose of DAs in prolactinoma patients was lower than in Parkinson’s patients, and conclusions from previous research on the relationship between DAs dose and the occurrence of psychological side effects in patients with pituitary prolactinoma were inconsistent. Similar to previous case reports and studies [23, 26, 27], we found that the parameters related to DA treatment, including duration of treatment, cumulative dose, and maximum daily dose, had no significant correlation with the occurrence of side effects. In 2007, Davie [27] et al. first reported a case of prolactinoma in women who developed pathological gambling after 1 year of treatment with a low dose of DAs (cabergoline, 0.25 mg per week). Later, De Sousa [23] reported 8 prolactinoma patients with hypersexuality after continuous low-dose DAs treatment. Additionally, patients with ICD screening by the scale from the Mayo Clinic (as shown in Table 3) received bromocriptine with a minimum–maximum daily dose of 2.5 mg, a minimum duration of 6 months, and a minimum cumulative dose of 450 mg, suggesting that ICDs may also occur with a lower dose of DAs. Therefore, the occurrence of side effects in pituitary prolactinoma patients treated with DAs in this study may not be significantly dose-dependent.

This study focused on the physical and psychological side effects of DAs in Chinese patients with prolactinoma, but it has some limitations. First, the causal relationship between physical and psychological symptoms and DAs treatment was uncertain because of a cross-sectional study. Second, the number of subjects in each group was relatively small, and further testing with a larger sample size is probably needed. Third, additional factors such as social and cultural influences may contribute to psychosocial symptoms, and we did not evaluate any of these. Finally, this study was only from one center, and multicenter surveys may be required in the future in order to validate these initial results.

## Conclusion

This study is the first to measure the physical and psychological side effects of DAs therapy on Chinese patients with pituitary prolactinoma. We found that there was a low incidence for ICDs in Chinese prolactinoma patients receiving DAs treatment but the physical symptoms were common, with an incidence of 51.0%. However, we found no significant correlation between DAs treatment parameters and the occurrence of side effects. Further multicenter surveys and larger sample size from future studies may be needed to validate these results.

## Supplementary Information

**Additional file 1.** Physical symptoms relatedto side effects of dopamine agonist drugs.

## Abbreviations

DAs: Dopamine receptor Agonist drugs
ICDs: Impulsive Control Disorders
BIS-11: Barratt Impulsivity Scale-11
PRL: Prolactin
MIDI: Minnesota Interview Questionnaire
MIDI-R: a revised version of Minnesota Impulsive Disorders Interview Questionnaire

## Declarations

### Availability of data and materials

The data in the current study are available from the corresponding author upon reasonable request.

## References

[1] Maiter D (2019). Management of Dopamine Agonist-Resistant Prolactinoma. Neuroendocrinology.

[2] Melmed S (2020). Pituitary-Tumor Endocrinopathies. N Engl J Med.

[3] Ben-Jonathan N, Hnasko R (2001). Dopamine as a prolactin (PRL) inhibitor. Endocr Rev.

[4] Beaulieu JM, Gainetdinov RR (2011). The physiology, signaling, and pharmacology of dopamine receptors. Pharmacol Rev.

[5] Souteiro P, Belo S, Carvalho D (2020). Dopamine agonists in prolactinomas: when to withdraw?. Pituitary.

[6] Ananthakrishnan S. The Dark Side to Dopamine Agonist Therapy in Rolactinoma Management. Endocr Pract. 2017. 10.4158/EP161709.CO. PMID:28156150. 10.4158/EP161709.CO 28156150

[7] Ioachimescu AG, Fleseriu M, Hoffman AR, Vaughan Iii TB, Katznelson L (2019). Psychological effects of dopamine agonist treatment in patients with hyperprolactinemia and prolactin-secreting adenomas. Eur J Endocrinol.

[8] Tran T, Brophy JM, Suissa S, Renoux C (2015). Risks of Cardiac Valve Regurgitation and Heart Failure Associated with Ergot- and Non-Ergot-Derived Dopamine Agonist Use in Patients with Parkinson's Disease: A Systematic Review of Observational Studies. CNS Drugs.

[9] Bancos I, Nannenga MR, Bostwick JM, Silber MH, Erickson D, Nippoldt TB (2014). Impulse control disorders in patients with dopamine agonist-treated prolactinomas and nonfunctioning pituitary adenomas: a case-control study. Clin Endocrinol.

[10] Hinojosa-Amaya JM, Johnson N, González-Torres C, Varlamov EV, Yedinak CG, McCartney S (2020). Depression and Impulsivity Self-Assessment Tools to Identify Dopamine Agonist Side Effects in Patients With Pituitary Adenomas. Front Endocrinol (Lausanne)..

[11] Dogansen SC, Cikrikcili U, Oruk G, Kutbay NO, Tanrikulu S, Hekimsoy Z (2019). Dopamine Agonist-Induced Impulse Control Disorders in Patients With Prolactinoma: A Cross-Sectional Multicenter Study. J Clin Endocrinol Metab.

[12] Chanson P, Maiter D (2019). The epidemiology, diagnosis and treatment of Prolactinomas: The old and the new. Best Pract Res Clin Endocrinol Metab..

[13] Chinese Pituitary adenoma Cooperative Group (2014) (2014). Consensus on diagnosis and treatment of pituitary prolactin adenoma in China (2014 edition). Natl Med J China.

[14] Endocrinology Group (2016). Society of Obstetrics and Gynecology, Chinese Medical Association. Consensus on diagnosis and treatment of hyperprolactinemia in women. Chin J Obstet Gynecol.

[15] Melmed S, Casanueva FF, Hoffman AR, Kleinberg DL, Montori VM, Schlechte JA (2011). Diagnosis and Treatment of Hyperprolactinemia: An Endocrine Society Clinical Practice Guideline. J Clin Endocrinol Metab.

[16] Webster J (1996). A comparative review of the tolerability profiles of dopamine agonists in the treatment of hyperprolactinaemia and inhibition of lactation. Drug Saf.

[17] Webster J, Piscitelli G, Polli A, Ferrari CI, Ismail I, Scanlon MF (1994). A comparison of cabergoline and bromocriptine in the treatment of hyperprolactinemic amenorrhea. Cabergoline Comparative Study Group. N Engl J Med.

[18] Noronha S, Stokes V, Karavitaki N, Grossman A (2016). Treating prolactinomas with dopamine agonists: always worth the gamble?. Endocrine.

[19] Martinkova J, Trejbalova L, Sasikova M, Benetin J, Valkovic P (2011). Impulse control disorders associated with dopaminergic medication in patients with pituitary adenomas. Clin Neuropharmacol.

[20] Celik E, Ozkaya HM, Poyraz BC, Saglam T, Kadioglu P (2018). Impulse control disorders in patients with prolactinoma receiving dopamine agonist therapy: a prospective study with 1 year follow-up. Endocrine.

[21] De Sousa SMC, Baranoff J, Rushworth RL, Butler J, Sorbello J, Vorster J (2020). Impulse Control Disorders in Dopamine Agonist-Treated Hyperprolactinemia: Prevalence and Risk Factors. J Clin Endocrinol Metab.

[22] Weintraub D, Claassen DO (2017). Impulse Control and Related Disorders in Parkinson's Disease. Int Rev Neurobiol.

[23] De Sousa SM, Chapman IM, Falhammar H, Torpy DJ (2017). Dopa-testotoxicosis: disruptive hypersexuality in hypogonadal men with prolactinomas treated with dopamine agonists. Endocrine.

[24] Weintraub D, Koester J, Potenza MN, Siderowf AD, Stacy M, Voon V (2010). Impulse control disorders in Parkinson disease: a cross-sectional study of 3090 patients. Arch Neurol.

[25] Bastiaens J, Dorfman BJ, Christos PJ, Nirenberg MJ (2013). Prospective cohort study of impulse control disorders in Parkinson's disease. Mov Disord.

[26] Falhammar H, Yarker JY (2009). Pathological gambling and hypersexuality in cabergoline-treated prolactinoma. Med J Aust.

[27] Davie M (2007). Pathological gambling associated with cabergoline therapy in a patient with a pituitary prolactinoma. J Neuropsychiatry Clin Neurosci.

