# Supplementary material for: The side effects of dopamine receptor agonist drugs in Chinese prolactinoma patients: a cross sectional study
Source: BMC Endocr Disord. 2022 Apr 11;22:97. doi: 10.1186/s12902-022-01009-3 (PMC9004168; doi:10.1186/s12902-022-01009-3)
Supplement: Supplementary file 1 — Additional file 1. Physical symptoms relatedto side effects of dopamine agonist drugs. [file 12902_2022_1009_MOESM1_ESM.docx]

**Physical symptoms related to side effects of dopamine agonist drugs**

- Systemic and cutaneous symptoms

🗆 Edema

🗆 Asthenia

🗆 Fatigue

🗆 Skin bruising

🗆 None

🗆 Others

- Respiratory symptoms

🗆 Chest pain

🗆 Chest distress

🗆 Dyspnea

🗆 Tussiculation

🗆 None

🗆 Others

- Gastrointestinal symptoms

🗆 Dry mouth

🗆 Loss of appetite

🗆 Nausea

🗆 Vomiting

🗆 Hepatic insufficiency

🗆 Constipation

🗆 diarrhea

🗆 None

🗆 Others

- Cardiovascular symptoms

🗆 Orthostatic hypotension

🗆 Arrhythm

🗆 Heart valve insufficiency

🗆 Worse angina

🗆 None

🗆 Others

- Psychological and neurological symptoms symptoms

🗆 Headache

🗆 Dizzy

🗆 Memory loss

🗆 Hallucination

🗆 Paraesthesia

🗆 Narcolepsy

🗆 Somnolence

🗆 Perversion of sleep

🗆 None

🗆 Others

- Renal symptoms

🗆 Renal insufficiency

🗆 None

🗆 Others
